# Exposure of RML scrapie agent to a sodium percarbonate-based product and sodium dodecyl sulfate renders PrP^Sc^ protease sensitive but does not eliminate infectivity

**DOI:** 10.1186/1746-6148-9-8

**Published:** 2013-01-11

**Authors:** Jodi D Smith, Eric M Nicholson, Gregory H Foster, Justin J Greenlee

**Affiliations:** 1 Virus and Prion Research Unit, National Animal Disease Center, USDA, Agricultural Research Service, 1920 Dayton Ave, Ames, IA 50010, USA

**Keywords:** Inactivation, Prion, Scrapie, Sodium dodecyl sulfate, Sodium percarbonate

## Abstract

**Background:**

Prions, the causative agents of the transmissible spongiform encephalopathies, are notoriously difficult to inactivate. Current decontamination recommendations by the World Health Organization include prolonged exposure to 1 N sodium hydroxide or > 20,000 ppm sodium hypochlorite, or autoclaving. For decontamination of large stainless steel surfaces and equipment as in abattoirs, for example, these methods are harsh or unsuitable. The current study was designed to evaluate the effectiveness of a commercial product containing sodium percarbonate to inactivate prions. Samples of mouse brain infected with a mouse-adapted strain of the scrapie agent (RML) were exposed to a sodium percarbonate-based product (SPC-P). Treated samples were evaluated for abnormal prion protein (PrP^Sc^)-immunoreactivity by western blot analysis, and residual infectivity by mouse bioassay.

**Results:**

Exposure to a 21% solution of SPC-P or a solution containing either 2.1% or 21% SPC-P in combination with sodium dodecyl sulfate (SDS) resulted in increased proteinase K sensitivity of PrP^Sc^. Limited reductions in infectivity were observed depending on treatment condition. A marginal effect on infectivity was observed with SPC-P alone, but an approximate 2–3 log_10_ reduction was observed with the addition of SDS, though exposure to SDS alone resulted in an approximate 2 log_10_ reduction.

**Conclusions:**

This study demonstrates that exposure of a mouse-adapted scrapie strain to SPC-P does not eliminate infectivity, but does render PrP^Sc^ protease sensitive.

## Background

The transmissible spongiform encephalopathies (TSE) include diseases such as scrapie in sheep and goats, bovine spongiform encephalopathy (BSE) in cattle, and Creutzfeldt-Jakob disease (CJD) in humans. Prions are the causative agent of TSEs and consist predominantly of an abnormally folded, partially protease resistant isoform of the cellular prion protein (termed PrP^Sc^) [1]. Prions are notoriously difficult to inactivate and, in the case of BSE, pose a lethal zoonotic disease risk [2,3]. Oral exposure is considered the natural route of infection for most TSEs affecting domestic species, and is considered the route by which humans acquired a new variant form of CJD (vCJD) after exposure to BSE-contaminated beef during the BSE epidemic in the United Kingdom [4]. Effective prion decontamination methods in settings such as abattoirs, for example, are desirable to further minimize the risk of zoonotic TSE transmission. Efficacious decontamination procedures that can be applied to environmental settings also are desirable to aid in the control of TSEs such as scrapie and chronic wasting disease, which are horizontally transmitted [5,6].

Prions are unusually resistant to methods effective at inactivating conventional microorganisms, such as moderate heating, ultraviolet irradiation, and formalin exposure [7]. Current decontamination recommendations by the World Health Organization, depending on material to be sterilized, include autoclaving at 134°C for up to 1 hour, or prolonged exposure to 1 N sodium hydroxide or ≥ 20,000 ppm sodium hypochlorite [8]. Procedures such as treatment with sodium hydroxide or sodium hypochlorite are especially detrimental to delicate surgical and diagnostic equipment, spurring research into less caustic alternatives. Recent lines of investigation have included treatment of contaminated material with proteolytic enzymes [9,10], sodium dodecyl sulfate [11,12], and peroxygen compounds [13-15] with variable success. A 4.5-5.6 log_10_ reduction in infectivity of scrapie strain 263 K bioassayed in Syrian hamsters was demonstrated after treatment of contaminated stainless steel wires with vaporized hydrogen peroxide with or without an enzymatic cleaner [15]. In contrast, a ≤1 log_10_ reduction in infectivity was demonstrated with liquid hydrogen peroxide [13].

Sodium percarbonate, an oxidizing agent comprising an adduct of sodium carbonate and hydrogen peroxide (2 Na_2_CO_3_ · 3 H_2_O_2_), is the active ingredient in a number of commercially available cleaning products. A major advantage of sodium percarbonate is its high degree of environmental compatibility, with degradation products consisting of water, oxygen, and sodium carbonate. In aqueous solution, sodium percarbonate generates a pH of 10–11. Prompted by positive results using vaporized hydrogen peroxide to inactivate the scrapie agent, we investigated the prion-inactivating potential of a commercial product containing sodium percarbonate (SPC-P). Sodium dodecyl sulfate alone has been shown to variably reduce prion infectivity [11,16,17]. We chose to incorporate it in this study due to its detergent and denaturant properties. Brain homogenate from terminally ill C57BL/6 mice positive for the mouse-adapted RML strain of scrapie was subjected to various SPC-P-based treatment conditions. Western blot (WB) analysis was used to detect residual PrP^Sc^ in treated samples, and residual infectivity was assayed by intracranial inoculation into prion protein overexpressing *tg*a*20* mice [18].

## Results

### Immunoblotting

**Figure 1:**
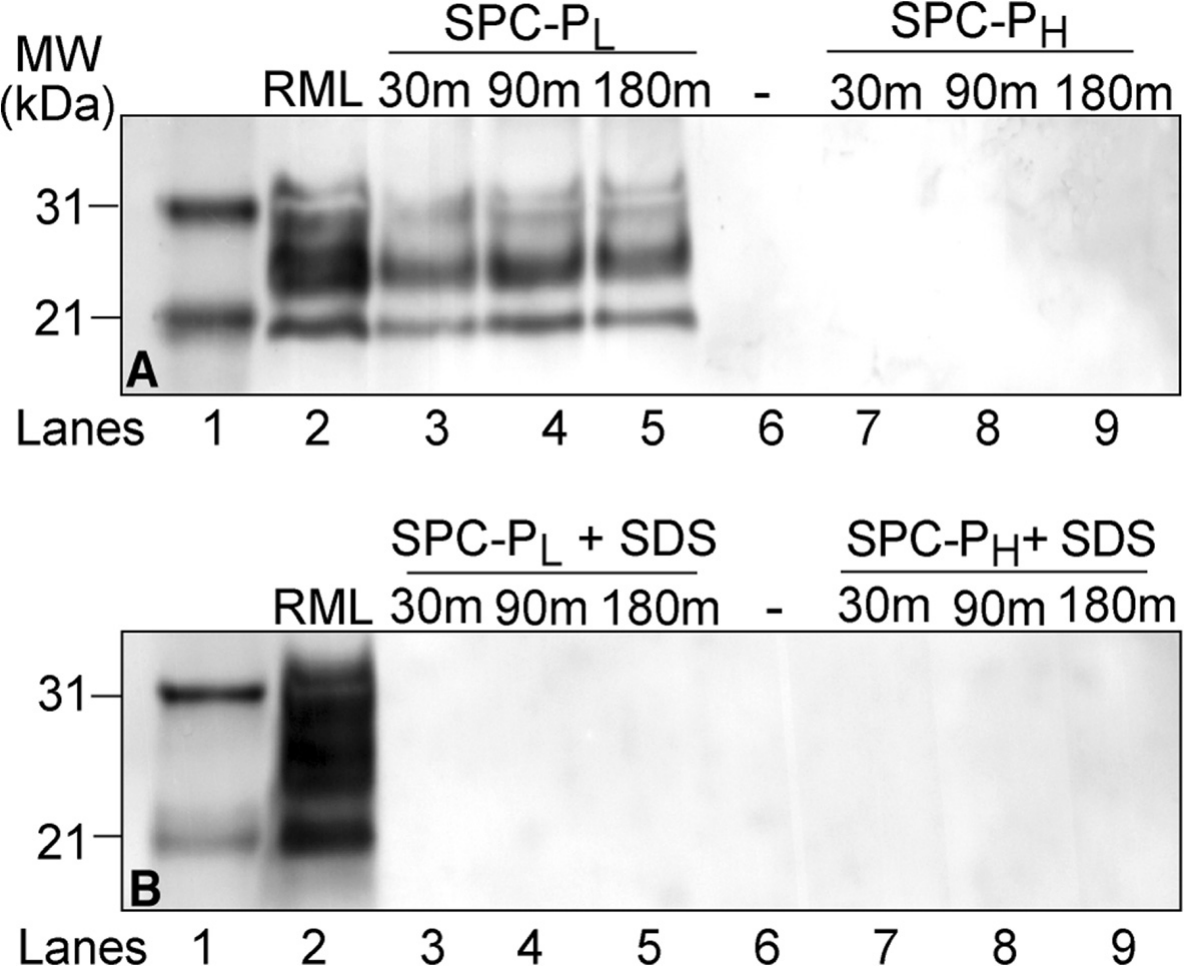
**Western blot of PrP**^**Sc**^**in brain homogenate from RML scrapie-affected C57Bl/6 mice treated with SPC-P**_**L**_**or SPC-P**_**H**_**alone (A) or in combination with 2.5% SDS (B).A**) PrP^Sc^ was still detectable after PK digestion of samples exposed to SPC-P_L_ (lanes 3–5), but not in samples treated with SPC_H_ (lanes 7–9). **B**) PrP^Sc^ immunoreactivity was undetectable after PK digestion of samples exposed to either SPC-P_L_ + SDS (lanes 3–5) or SPC-P_H_ + SDS (lanes 7–9). Lane 1, molecular weight marker; lane 2, RML positive control; lane 6, empty. Abbreviations: MW, molecular weight; SPC-P_L_ or _H_, low or high concentration sodium percarbonate-based product; SDS, sodium dodecyl sulfate; m, minutes.

**Figure 2:**
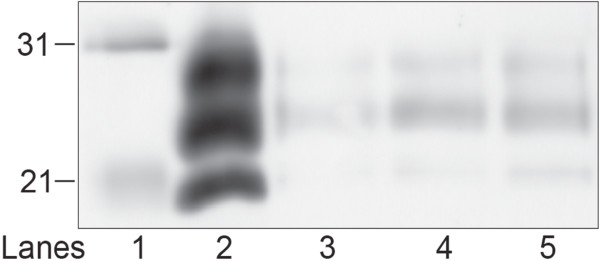
**Western blot of PrP**^**Sc**^**in brain homogenate from RML scrapie-affected C57Bl/6 mice treated with 2.5% SDS for 30, 90, or 180 minutes.** PrP^Sc^-immunoreactivity was decreased but still detectable after PK digestion of samples exposed to SDS for 30 (lane 3), 90 (lane 4), or 180 minutes (lane 5). Lane 1, molecular weight marker (kDa); lane 2, RML positive control.

**Figure 3:**
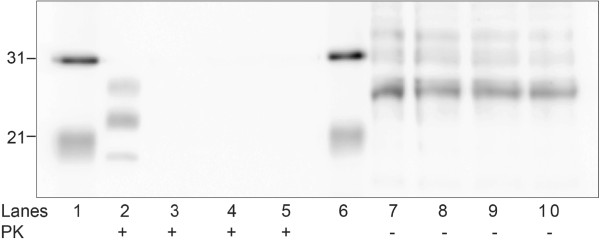
**Western blot of PrP**^**Sc**^**in brain homogenate from RML scrapie-affected C57Bl/6 mice treated with 0.35 M sodium hydrogen phosphate buffered solution (pH 11) for 30 (lanes 3, 8), 90 (lanes 4, 9), or 180 minutes (lanes 5, 10).** PrP^Sc^-immunoreactivity was undetectable after PK digestion (lanes 3–5). PK digestion was omitted prior to immunoblotting for samples in lanes 7–10. Lanes 1 and 6, molecular weight marker (kDa); lanes 2 and 7, RML positive control.

Residual PrP^Sc^ in brain homogenate treated with a low or high concentration of the SPC-containing product (SPC-P_L_ and SPC-P_H_, respectively) alone or in combination with 2.5% SDS was assayed via WB. Immunoblots were performed in triplicate with representative blots presented in Figure 1. Immunoreactivity for the di-, mono-, and unglycosylated forms of PrP^Sc^ was present in SPC-P_L_ treated samples after proteinase K (PK) digestion (Figure 1A, lanes 3–5), but was undetectable in SPC-P_H_ treated samples (Figure 1A, lanes 7–9). When PK digestion was omitted, PrP immunoreactivity was similarly detectable in all SPC-P_L_ treated samples and faint banding was present in SPC-P_H_ treated samples (data not shown). When samples treated with either SPC-P_L_ or SPC-P_H_ in combination with SDS were subjected to limited proteolysis with PK prior to immunoblotting, PrP^Sc^ immunoreactivity was undetectable for all treatment conditions (Figure 1B, lanes 3–5 and 7–9). RML brain homogenate exposed to SDS only retained detectable but decreased PrP^Sc^ immunoreactivity after PK digestion (Figure 2). Both concentrations of SPC-P used in this study generated a pH of approximately 11 in solution. To evaluate the effect of pH, brain samples buffered with 0.35 M sodium hydrogen phosphate (pH 11) were incubated at room temperature for 30, 90, or 180 minutes. PrP^Sc^ immunoreactivity was undetectable after PK digestion at all time points (Figure 3).

### Mouse bioassay

Residual infectivity in treated samples was assayed via intracranial inoculation of *tg*a*20* mice. The average number of days to terminal disease for the positive control group (untreated RML-positive brain homogenate) was 65.5 ± 3.4 days, which is typical of RML disease kinetics in *tg*a*20* mice inoculated intracranially [18]. Ten-fold serial dilutions of this stock resulted in an increase in incubation and mean survival times. The survival curves generated from these data were used as the comparative standard when evaluating survival time in mice inoculated with treated samples and for approximating reductions in infectivity. All animals inoculated with a 10^-5^ or greater dilution survived until termination of the study at 15 months post-inoculation (PI) (Table 1), with the exception of 10 mice removed from the study and censored from survival statistics due to intercurrent disease. One mouse from the 10^-4^ group was also censored from survival data due to intercurrent disease. Consistent clinical signs observed in scrapie-affected mice included ataxia that progressed to a listing or rolling gait in some cases, pelvic limb paresis, and lethargy. All but 2 animals in the negative control group (treated RML-negative brain homogenate) survived until study termination at 15 months PI. One mouse was euthanized due to severe hydrocephalus and the other death was undetermined.

**Table 1:** **Survival times of *tg*a*20 *mice inoculated intracranially with serially diluted or treated RML brain homogenate**

**Dilution or treatment**	**Mean survival time in days ± SD (% survival)**
1) RML titration series^*^	
10^0^	65.5 ± 3.4 (0%)
10^-1^	73.6 ± 3.3 (0%)
10^-2^	83.2 ± 6.1 (0%)
10^-3^	195.4 ± 148.5 (22%)
10^-4^	394.8 ± 120.3 (78%)
10^-5^ through 10^-12^	455 (100%) – study termination
*2) Treated RML*	
SPC-P_L_ x 30 min	67.1 ± 2.6 (0%)
SPC-P_L_ x 90 min	68.8 ± 4.6 (0%)
SPC-P_L_ x 180 min	68.5 ± 2.8 (0%)
SPC-P_H_ x 30 min	70.3 ± 6.0 (0%)
SPC-P_H_ x 90 min	71.0 ± 5.1 (0%)
SPC-P_H_ x 180 min	73.8 ± 3.2 (0%)
SPC-P_L_ + SDS x 30 min	93 ± 9.7 (0%)
SPC-P_L_ + SDS x 90 min	136.4 ± 72.2 (10%)
SPC-P_L_ + SDS x 180 min	178.7 ± 92.4 (20%)
SPC-P_H_ + SDS x 30 min	92.5 ± 7.6 (0%)
SPC-P_H_ + SDS x 90 min	86.4 ± 6.9 (0%)
SPC-P_H_ + SDS x 180 min	84.7 ± 11.1 (0%)
SDS x 30 min	84.9 ± 12.3 (0%)
SDS x 90 min	107.5 ± 40.5 (0%)
SDS x 180 min	85.7 ± 6.7 (0%)

Abbreviations: *SD*, standard deviation; *SPC-P*_*L*_ or _*H*_, low or high concentration sodium percarbonate-based product; SDS, sodium dodecyl sulfate.

^*^1% w/v starting (10^0^) concentration.

**Figure 4:**
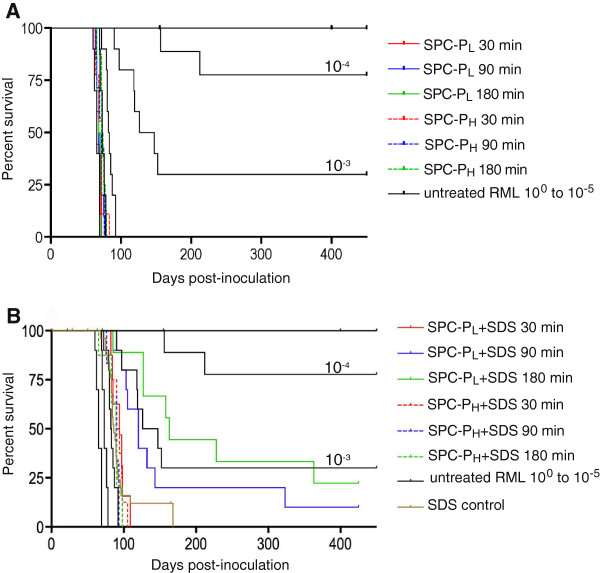
**Effect of SPC-P with or without SDS on infectivity.** Kaplan-Meier survival curves were generated to compare SPC-P treatment conditions with 10-fold serial dilutions of RML scrapie in *tg*a*20* mice. **A**) Treatment with SPC-P_L_ or SPC-P_H_ alone had minimal effect on infectivity with 0% survival. **B**) Combining SPC-P and SDS resulted in 2–3 log_10_ reductions in infectivity depending on treatment condition. Greater reductions and higher survival percentages were observed with the SPC-P_L_ + SDS groups in a time-dependent manner. Exposure to SDS alone resulted in an approximate 2 log_10_ reduction in infectivity (combined data from 30 min., 90 min., and 180 min. exposure groups). Abbreviations: SPC-P_L_ or _H_, low or high concentration sodium percarbonate-based product; SDS, sodium dodecyl sulfate.

Treatment of brain homogenate with SPC-P_L_ or SPC-P_H_ alone had minimal effect on infectivity. The average time to disease in all animals inoculated with SPC-P_L_ or SPC-P_H_ was 68.2 ± 3.4 days or 71.7 ± 5.0 days, respectively. The limited increase in survival time of the SPC-P_H_ treated groups corresponded to an approximate 1 log_10_ reduction in infectivity (Figure 4A). In contrast, exposure of the inoculum to a solution of SPC-P_L_ or SPC-P_H_ containing SDS resulted in a 2–3 log_10_ reduction in infectivity (Figure 4B). The average time to terminal disease for the SPC-P_H_ + SDS groups at 30 min, 90 min, and 180 min was 92.5 ± 7.6, 86.4 ± 6.9, and 84.7 ± 11.1 days, respectively. These values corresponded to an approximate 2 log_10_ reduction; however, the transmission rate was 100%. A 2–3 log_10_ reduction was achieved with SPC-P_L_ + SDS. This effect was time-dependent with 30 min, 90 min, and 180 min treatment groups surviving on average 93 ± 9.7, 136.4 ± 72.2, and 178.7 ± 92.4 days, respectively. Additionally, 10% of mice in the 90 min treatment group and 20% in the 180 min group survived until study termination at 15 months PI. Mice inoculated with samples exposed to SDS alone for 30 min, 90 min, or 180 min developed disease in 84.9 ± 12.3, 107.5 ± 40.5, and 85.7 ± 6.7 days, respectively, corresponding to an approximate 2 log_10_ reduction in infectivity for each group (Figure 4B), but with a transmission rate of 100%. Eight mice total were censored from survival statistics due to intercurrent disease including 5 mice in the SPC-P_H_ + SDS group (1 due to complications from inoculation, 1 death undetermined, 3 euthanized due to severe hydrocephalus), 2 mice in the 30 min SPC-P_L_ + SDS group (cause of death undetermined), and 1 mouse in the 180 min SPC-P_L_ + SDS group (euthanized due to incisor malocclusion).

### Neuropathology and PrP immunohistochemistry

Lesions of spongiform encephalopathy (SE) in affected mice were predominantly present in thalamic and midbrain nuclei and consisted of 7 – 20 μm, clear, round vacuoles primarily within the neuropil, but also occasionally present within neurons. Milder SE lesions were present in the cerebral cortex, septal nuclei, cornus ammonis regions of the hippocampus, cerebellar nuclei, and brainstem nuclei. The cerebellar cortex lacked definitive SE lesions as did cerebral and cerebellar white matter. Mild to moderate gliosis and scattered degenerate neurons, characterized by hyperchromasia, a shrunken and angular profile, and loss of fine nuclear detail were associated with areas of SE. Approximately 8% of mice exposed to inocula from the SPC-P + SDS treated groups developed varying degrees of hydrocephalus that was most often noted incidentally at necropsy. Immunoreactivity for PrP^Sc^ in affected mice corresponded to areas of SE and was most intense in midbrain and vestibular nuclei. Immunoreactivity was predominantly present as fine punctate granules within the neuropil and less often as fine deposits around individual neuronal soma and dendrites (perineuronal or synaptic).

## Discussion

In this report, we investigated the effectiveness of a product containing the oxidizing agent sodium percarbonate to inactivate the RML scrapie agent. Sodium dodecyl sulfate at 2.5% w/v also was added to certain treatment groups to evaluate the combinatorial effect of SPC-P and SDS. Treated samples were evaluated for PrP^Sc^ immunoreactivity by western blot and residual infectivity by mouse bioassay using *tg*a*20* mice. Our choice for using the *tg*a*20* mouse for the bioassay was largely due to the well-characterized and relatively rapid disease kinetics of RML scrapie in this strain of mouse [18]. We were able to measure a reduction in infectivity over a range of 10^5^-fold with this model, which was adequate for this particular study because of the poor efficacy of the product resulting in survival times well within the detection range. Product choice and treatment conditions for this study were defined by the following considerations: 1) the product should be sufficiently non-hazardous to the environment and human health to be applied safely on a large-scale; and 2) treatment conditions (e.g. time and temperature) should be similar to those that could be realistically achieved in, for example, an abattoir.

A major finding of this study was the increased sensitivity of PrP^Sc^ to PK by the SPC-based product without (SPC-P_H_ only) or with SDS at room temperature, as judged by immunoblotting after exposure of the samples to limited proteolysis. Based on the loss of detectable PrP^Sc^ immunoreactivity after incubation at pH 11, it appears this effect may be largely pH-dependent. It is well established that prion infectivity is reduced under extremely basic conditions, such as exposure to NaOH (pH 12–14) [19-21]. While the pH generated by SPC-P is lower at 11, it appears to be a favorable characteristic of the compound with regard to PrP^Sc^ protease sensitivity. However, a solely pH-dependent effect does not explain why SPC-P_L_ treatment alone (pH 11) did not yield similar WB results. One possible explanation is that a lower concentration of the product may have contained diminished buffering capacity resulting in a drop in pH as treatment proceeded, but serial pH evaluation of treated brain homogenate at 30, 90, and 180 min revealed that the pH remained above 10.7. Although treatment with the SPC product did render PrP^Sc^ sensitive to digestion by proteinase K, it did not eliminate infectivity. Recent studies examining prion infectivity in infected tissue and cell cultures have also demonstrated loss of detectable PrP^Sc^ on western blot, but residual infectivity [22,23]. Our results support the inference that biochemical analysis alone is insufficient for determination of prion infectivity. The observed PrP^Sc^/infectivity mismatch in this study and in others warrants a number of considerations including WB sensitivity, epitope disruption by inactivation treatments, and alternative infectious agents to PrP^Sc^, such as PK-sensitive forms of PrP or viruses. It is possible the amount of residual PrP^Sc^ in our treated samples was below the detection limit of our WB (0.025 mg equivalents of brain tissue for this particular inoculum [24]), or it may be that a true dissociation of PrP^Sc^ and TSE infectivity exists supporting the actuality of alternative infectious agents to PrP^Sc^[25]. A recent study has demonstrated poor correlation between infectivity and WB results for sheep scrapie and sheep BSE [26] in line with observations that PK-sensitive PrP particles are associated with disease [27,28].

The bioassay results we present indicate that exposure to the selected SPC-based product alone or in combination with 2.5% SDS is not a viable option for the inactivation of prions. No decrease in infectivity was observed using the SPC-P_L_ solution alone, and a modest 1 log_10_ reduction was achieved with the SPC-P_H_ solution. However, recent investigations have demonstrated differential susceptibility of distinct prion strains to the same inactivation procedure [29]; therefore, we are currently investigating the efficacy of these treatment conditions in an ovine scrapie model. It should also be acknowledged that chemical treatment of the scrapie agent has been shown to delay the dose–response relationship [30,31] resulting in prolonged incubation times without a change in calculable titer. It is possible our results could be reflecting this phenomenon, but without bioassay data from serial dilutions of treated brain homogenate this cannot be definitively determined. Some caution may therefore be warranted when interpreting these results. The addition of 2.5% SDS to the SPC-P solutions resulted in a 2–3 log_10_ reduction in infectivity, but exposure to SDS alone resulted in an approximate 2 log_10_ reduction. This suggests much of the observed combinatorial effect was due to SDS. Prior studies using SDS have demonstrated minimal effects on CJD infectivity [16], but up to a 3 log_10_ reduction on scrapie infectivity [17]. Exposure of hamster-adapted Sc237 scrapie to room temperature SDS at pH values of ≤4.5 or ≥10 resulted in increased PK sensitivity of PrP^Sc^, and exposure to acidic SDS resulted in decreased infectivity [11]. Since SDS at room temperature is an effective denaturant at a pH ≥10, this could have contributed to the loss of detectable PrP^Sc^-immunoreactivity we observed after proteolysis in samples treated with SPC-P and SDS. There was also enhanced reduction in infectivity with the combination of SPC-P_L_ and SDS. This may be indicative of an enhanced effect of SDS under basic conditions or a two-step mechanism whereby denaturation of PrP^Sc^ by the relatively high pH of the solution and/or SDS is followed by exposure of sites sensitive to oxidative damage. Alternatively, the two treatment components could be acting on different PrP^Sc^ fractions in the inoculum resulting in an additive effect since the combination of SPC-P_L_ and SDS was roughly equivalent to slightly greater than the sum of the effects of each individual component. The combination of SPC-P_H_ and SDS did not provide an equivalent or better increase in survival time than the combination of SPC-P_L_ and SDS. While we are confident in this result, we cannot definitively explain this observation. Perhaps disease in this group was exacerbated by oxidative damage induced by the introduction of treated brain samples containing a greater concentration of sodium percarbonate. Oxidative stress, whether a cause or consequence of disease progression, is considered an important contributor to prion neuropathology [32-34]. It is also possible that the SPC-P solution at higher concentration may somehow be interfering with the denaturing action of SDS. SDS action may be enhanced when combined with lower concentrations of SPC-P for longer exposure times, but restricted by higher concentrations, perhaps via chemical modification of SDS binding sites on the protein.

Oxidizing agents have been used with variable success in prion inactivation studies. Exposure of prions to halogens such as sodium hypochlorite at ≥ 20,000 ppm is an accepted means of decontamination [8], but chlorine dioxide is much less effective at inactivating hamster-adapted 263 K scrapie [35]. Peroxygens such as liquid hydrogen peroxide [13,35,36] and peracetic acid [37] also promote limited inactivation. However, recent studies using vaporized hydrogen peroxide to decontaminate stainless steel surfaces have demonstrated significant reductions in infectivity for hamster-adapted 263 K scrapie and mouse-adapted BSE [13,15]. A protective effect from oxidation by peracetic acid has been demonstrated with the ME7 scrapie agent and attributed to prion aggregation [37]. Peracetic acid at 2% was effective at inactivating the ME7 scrapie agent in intact brain tissue, but not homogenized tissue. Samples in the current study were homogenized, which may have imparted a degree of protection from oxidation and contributed to the ineffectiveness of SPC-P alone at decreasing infectivity. We propose that the addition of SDS would have decreased aggregation of cell membranes to which infectivity is bound, thus enhancing the activity of SPC-P and perhaps contributing to the increased survival observed with the combination.

## Conclusions

This study demonstrates that exposure of the RML scrapie agent to an SPC-containing product alone or in combination with SDS does not eliminate prion infectivity, but does render PrP^Sc^ sensitive to proteinase K. Because of this, it is interesting to consider the potential viability of a combination of SPC and SDS, even at relatively low concentrations and mild temperatures, concomitant with or followed by a protease for prion decontamination. Also, because the SPC product we used contains additional proprietary ingredients, we cannot rule-out contributions to increased PK-sensitivity or increased survival by other components of the product. Studies in our laboratory are currently underway examining exposure of prions to chemical grade SPC with or without SDS followed by exposure to a protease.

## Methods

### Preparation of inoculum

A 10% w/v brain homogenate in PBS was prepared from a pool of clinically affected C57BL/6 mice inoculated with the mouse-adapted RML strain of scrapie [38] or uninfected C57BL/6 mice using a bead beater tissue homogenizer (Mini-Beadbeater-8, BioSpec). Briefly, brain tissue was placed inside a sealed polycarbonate tube along with PBS and a small volume of 1.0 mm silica beads. The sample was homogenized for 1 min at 4°C. This was repeated for a total of 5 homogenizations. Following centrifugation at 4,000 x G for 10 min at 4°C, the pellet was discarded and the sample was transferred to a new tube and stored at −80°C.

### Inactivation of inoculum

Brain samples from negative control or RML-positive mice were prepared as outlined above. Homogenates were diluted to a concentration of 5% in either a 2.1% (pH 10.7) or 21.0% (pH 10.6) solution of a commercial product containing SPC (OxiMagic, Clorox Company; 50-60% SPC) with or without 2.5% w/v SDS. The manufacturer’s instructions on concentration were followed (2.1% working solution), but additional parameters were experimentally defined. Samples were agitated under ambient oxygen in microcentrifuge tubes at 25°C for 30 min, 1.5 h, or 3 h, and then diluted with sterile saline to a final concentration of 1% for inoculation (final pH values of 10.3 and 10.6 for SPC-P_L_ and SPC-P_H_-treated samples, respectively). Samples were inoculated intracranially (see below) into 10 *tg*a*20* mice per treatment condition. Mice (n = 10/group) inoculated with RML-negative brain homogenate treated with 2.1% or 21.0% SPC-P with or without SDS served as negative controls. Mice (n = 10/group) inoculated with RML-positive brain homogenate incubated with 2.5% w/v SDS at 25°C for 30 min, 1.5 h, or 3 h were included as SDS-only controls. Samples were stored at −20°C until thawing for WB analysis and inoculation into *tg*a*20* mice. Mice were inoculated within 12–24 h of sample treatment.

### Immunoblotting

Treated samples from each inactivation condition were examined for PrP^Sc^ by WB. In addition, pH 11 control samples were prepared. For these control samples, RML-positive brain homogenate was incubated at room temperature for 30 min, 1.5 h, or 3 h in a 0.35 M sodium hydrogen phosphate buffered solution (pH 11). Pretreatment of brain homogenate with proteinase K (USB Corporation) was performed on one set of blots and omitted on repeated blotting of the same samples. Western blots were repeated three times with representative blots presented in Figure 1. Briefly, samples were digested with PK using a final concentration of 0.08 mg/mL at 48°C for 40 min. A proteinase inhibitor (Pefabloc, Roche) was added to a final concentration of 0.1 mg/ml to inhibit PK activity. Samples were dissolved in SDS-PAGE sample buffer and analyzed by standard WB procedures. A tissue equivalent of 1.0 mg was loaded onto the gel for each sample. PrP^Sc^ was detected using monoclonal antibody 6H4 (Prionics) at a 1:10,000 (0.1 μg/mL) dilution applied for 1 hour at room temperature or 4°C overnight. A biotinylated sheep anti-mouse secondary antibody and a streptavidin-horseradish peroxidase conjugate (GE Healthcare) were used in conjunction with a detection kit (ECL Plus, GE Healthcare) to detect immunolabeling. Secondary antibody and streptavidin-horseradish peroxidase incubations were conducted at room temperature for 1 h. Blots were developed using a Typhoon 9410 Variable Mode Imager (Molecular Dynamics).

### Mouse bioassay

To establish comparative survival curves, a 1% w/v RML brain homogenate was serially diluted (ten-fold dilutions; undiluted or 10^0^ through 10^-12^), and each dilution was intracranially inoculated into 10 mice of the B6;129S7-Prnp^tm1Cwe^Tg(Prnp)a20Cwe/CweCnrm (*tg*a*20*) mouse line [18]. For all inoculations, *tg*a*20* mice were anesthetized with isoflurane and a 30 gauge tuberculin syringe was used to inject 20 μL of brain homogenate into the right cerebral hemisphere at a depth of 3–5 mm. Mice were monitored for 48 hours post-inoculation for procedure-related adverse events. Mice were then monitored daily and euthanized when they displayed unequivocal neurological signs, or at the time of study termination (15 months PI). This experiment was carried out in accordance with the Guide for the Care and Use of Laboratory Animals (Institute of Laboratory Animal Resources, National Academy of Sciences, Washington, DC) and was approved by the National Animal Disease Center’s Animal Care and Use Committee (protocol #2422).

### Histopathology and immunohistochemistry

At necropsy, the skull cap was removed and the brain was extracted. A single parasagittal cut was made and two-thirds of the brain was immersed in 10% neutral buffered formalin and one-third was frozen. Fixed brain tissue was trimmed into 5 standard transverse sections [39] and tissues were processed by routine histological methods and embedded into paraffin blocks. Serial 4 μm sections were cut from the brain and stained with hematoxylin and eosin. For confirmation of scrapie infection, each brain was examined for characteristic spongiform lesions and/or PrP^Sc^-immunoreactivity by WB or immunohistochemistry. For immunohistochemistry, tissue sections were deparaffinized and rehydrated. Sections were autoclaved at 121°C for 20 min in distilled water and incubated for 5 min in 98% formic acid. Endogenous peroxidase activity was blocked with a 3% hydrogen peroxide solution. Sections were blocked in Tris buffered saline with 0.05% Tween 20, 1% bovine serum albumin, and 1.5% goat serum. Primary antibody (6H4 at 1:10,000) was applied for 1 hr. at room temperature. Secondary antibody with an HRP labeled polymer (Dako) was applied for 1 hr. at room temperature. Sections were incubated in a 3,3^′^-diaminobenzidine solution (Vector Laboratories), counterstained with hematoxylin, dehydrated, and cover-slipped.

### Statistical method

Kaplan-Meier survival curves were generated using statistical software (Prism version 4.0, GraphPad Software). To estimate reductions in infectivity, survival curves from treated groups were compared to those of the titration study using the logrank test with a level of statistical significance of 0.05. Mice that died within the first 3 weeks PI due to complications related to intracranial inoculation or were removed from the study for humane reasons (e.g. disease unrelated to TSE, injuries) prior to developing clinical signs were censored and not included in survival analyses.

## Abbreviations

BSE: Bovine spongiform encephalopathy; CJD: Creutzfeldt-Jakob disease; MW: Molecular weight; PI: Post-inoculation; PK: Proteinase K; PrP^Sc^: Abnormal or disease-associated isoform of the prion protein; RML: Rocky Mountain Laboratory strain of the scrapie agent; SDS: Sodium dodecyl sulfate; SE: Spongiform encephalopathy; SPC-P: Sodium percarbonate-based product; SPC-P_L_: Low concentration SPC-P; SPC-P_H_: High concentration SPC-P; TSE: Transmissible spongiform encephalopathy; WB: Western blot.

## Competing interests

The authors declare that they have no competing interests.

## Authors’ contributions

JDS conceived of the study, carried out the western blot and animal bioassay studies, and drafted the manuscript. EMN participated in the design of the study and interpretation of results. GHF carried out the end-point titration study. JJG participated in the design of the study and helped to draft the manuscript. All authors read and approved the final manuscript.

## Authors’ information

JDS and JJG are Research Veterinary Medical Officers in the NADC VPRU and diplomates of the American College of Veterinary Pathologists. JDS is a Postdoctoral Research Associate. EMN is a Research Chemist and Lead Scientist in the VPRU. GHF was a Postdoctoral Research Associate/Research Molecular Biologist in the VPRU during the time these experiments were performed.
